# Avian influenza A (H5N1) virus in dairy cattle: origin, evolution, and cross-species transmission

**DOI:** 10.1128/mbio.02542-24

**Published:** 2024-11-13

**Authors:** Ahmed Mostafa, Mahmoud M. Naguib, Aitor Nogales, Ramya S. Barre, James P. Stewart, Adolfo García-Sastre, Luis Martinez-Sobrido

**Affiliations:** 1 Department of Disease Intervention and Prevention, Texas Biomedical Research Institute, San Antonio, Texas, USA; 2 Center of Scientific Excellence for Influenza Viruses, National Research Centre, Giza, Egypt; 3 Department of Infection Biology and Microbiomes, University of Liverpool, Liverpool, United Kingdom; 4 Department of Medical Biochemistry and Microbiology, Uppsala University, Uppsala, Sweden; 5 Center for Animal Health Research, CISA-INIA-CSIC, Madrid, Spain; 6 Department of Microbiology, Icahn School of Medicine at Mount Sinai, New York, New York, USA; 7 Global Health and Emerging Pathogens Institute, Icahn School of Medicine at Mount Sinai, New York, New York, USA; 8 Department of Medicine, Division of Infectious Diseases, Icahn School of Medicine at Mount Sinai, New York, New York, USA; 9 The Tisch Cancer Institute, Icahn School of Medicine at Mount Sinai, New York, New York, USA; 10 Department of Pathology, Molecular and Cell-Based Medicine, Icahn School of Medicine at Mount Sinai, New York, New York, USA; 11 The Icahn Genomics Institute, Icahn School of Medicine at Mount Sinai, New York, New York, USA; The Ohio State University, Columbus, Ohio, USA

**Keywords:** avian viruses, cattle, highly pathogenic avian influenza virus, H5N1, United States

## Abstract

Since the emergence of highly pathogenic avian influenza virus (HPAIV) H5N1 of clade 2.3.4.4b as a novel reassortant virus from subtype H5N8, the virus has led to a massive number of outbreaks worldwide in wild and domestic birds. Compared to the parental HPAIV H5N8 clade 2.3.4.4b, the novel reassortant HPAIV H5N1 displayed an increased ability to escape species barriers and infect multiple mammalian species, including humans. The virus host range has been recently expanded to include ruminants, particularly dairy cattle in the United States, where cattle-to-cattle transmission was reported. As with the avian 2.3.4.4.b H5N1 viruses, the cattle-infecting virus was found to transmit from cattle to other contact animals including cats, raccoons, rodents, opossums, and poultry. Although replication of the virus in cows appears to be mainly confined to the mammary tissue, with high levels of viral loads detected in milk, infected cats and poultry showed severe respiratory disease, neurologic signs, and eventually died. Furthermore, several human infections with HPAIV H5N1 have also been reported in dairy farm workers and were attributed to exposures to infected dairy cattle. This is believed to represent the first mammalian-to-human transmission report of the HPAIV H5N1. Fortunately, infection in humans and cows, as opposed to other animals, appears to be mild in most cases. Nevertheless, the H5N1 bovine outbreak represents the largest outbreak of the H5N1 in a domestic mammal close to humans, increasing the risk that this already mammalian adapted H5N1 further adapts to human-to-human transmission and starts a pandemic. Herein, we discuss the epidemiology, evolution, pathogenesis, and potential impact of the recently identified HPAIV H5N1 clade 2.3.4.4b in dairy cattle in the United States. Eventually, interdisciplinary cooperation under a One Health framework is required to be able to control this ongoing HPAIV H5N1 outbreak to stop it before further expansion of its host range and geographical distribution.

## INTRODUCTION

**Fig 1:**
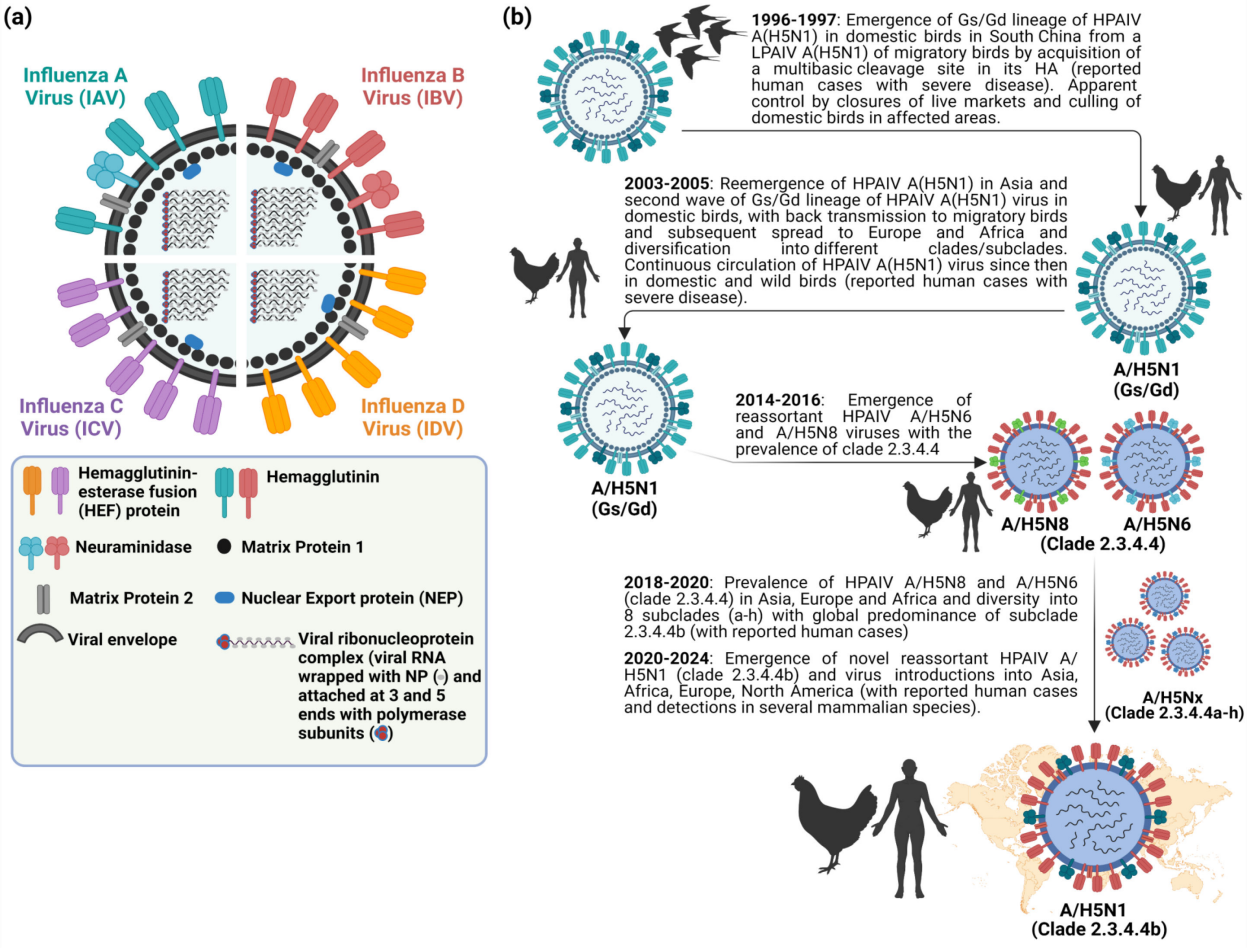
Viral particle composition and host range of IAV, IBV, ICV, and IDV: (a) Schematic diagram showing the structure of IAV, IBV, ICV, and IDV. IAV and IBV are made of eight viral segments with two surface glycoproteins: hemagglutinin (HA) and neuraminidase (NA). ICV and IDV contain seven viral segments with a single surface glycoprotein, the hemagglutinin-esterase-fusion (HEF) protein; (b) Documented history of highly pathogenic avian influenza virus (HPAIV) H5N1 emergence in migratory birds in 1996 and key evolutionary occurrences until 2024. The figure has been created/assembled with BioRender.com.

Influenza viruses are classified into four genera, namely *Alphainfluenzavirus* (influenza A virus [IAV]), *Betainfluenzavirus* (influenza B virus [IBV]), *Gammainfluenzavirus* (influenza C virus [ICV]) and *Deltainfluenzavirus* (influenza D virus [IDV]) (Fig. 1a). Seasonal epidemics in humans are caused annually due to infections with human IAV and IBV. In contrast to IBV, which is believed with some possible exceptions to be confined to humans, IAV is found to infect multiple animal species, including pigs, horses, marine mammals, dogs, bats, poultry, and several wild bird species. Infections with ICV could result in mild human illness, and it is not believed that they contribute to the main burden of severe respiratory disease in humans, as opposed to IAV and IBV. IDV infection occurs mainly in cattle, a natural reservoir with no reported cases in humans (1, 2). Nevertheless, evidence suggest that IDV can result in exposure of the workers due to occupational contact with infected cattle (3, 4). All influenza pandemics were caused by novel reassortants of IAV involving progenitor avian influenza viruses (AIV).

IAVs are further subtyped, based on the major surface glycoproteins hemagglutinin (HA) and neuraminidase (NA), into 19 antigenically different HA (H1–H19) and 11 distinct NA (N1–N11) subtypes (1, 5, 6). The HA protein hosts the major antigenic sites for each subtype. Variations to the antigenic sites are associated with the diversification of each HA subtype into clades or lineages that are antigenically distinct from the ancestor subtype (1). Based on their pathogenicity in chicken host, AIVs are classified into highly pathogenic (HPAIV), including some H5Nx and H7Nx viruses with multibasic cleavage site in their viral HA proteins, or low pathogenic strains including all AIV HA subtypes with monobasic cleavage site (1).

The RNA genome of IAV has a high mutational rate due to the lack of proofreading of its viral RNA-dependent RNA polymerase activity, making it error prone (7). Some of these acquired mutations at or around the vicinity of the antigenic sites are positively selected by IAV to gradually change their antigenic properties to escape adaptive immune responses to IAV strains circulating previously (antigenic drift) (8). These changes may also contribute to resistance to existing antiviral drugs, to facilitate viral entry, replication, or pathogenicity in the new host cells (9). In addition, the segmented nature of the IAV genome allows reassortment events when two or more IAVs infect the same cell, resulting in the emergence of novel IAV strain(s) with major changes in the antigenic properties (antigenic shift). Among the HPAIV strains, the H5N1 virus is frequently reported as the most pathogenic with significantly higher morbidity and mortality rates in avian species and mammals including humans (1, 10). Herein, we introduce a brief narrative of the widespread distribution of the emerging HPAIV H5N1 that led to multiple cross-species infections in wildlife and domestic animals, and the ongoing and unprecedented epizootic in dairy cattle.

## ECOLOGY AND EVOLUTION OF HPAIV H5N1 CLADE 2.3.4.4B

Since the emergence of highly pathogenic avian influenza virus (HPAIV) A/goose/Guangdong/1/1996 (Gs/Gd) H5N1 in China, the Gs/Gd lineage of HPAIV H5Nx has spread globally via migratory wild birds to infect various species and pose a threat to animal and human public health (1). Meanwhile, the H5-type Gs/Gd evolved into 10 distinct clades (0–9), and some of these clades are further categorized into different subclades (11). In late 2013, HPAIV H5N8 clade 2.3.4.4 emerged in China and shortly spread in early 2014 to South Korea and Japan (12). Later in 2014, the virus was further transmitted along different flyways to cause multiple outbreaks among poultry and wild birds in Asia, Europe, and North America (13). Meanwhile, the virus underwent multiple reassortment events with other AIV resulting in several H5Nx subtypes (e.g., H5N1, H5N2, H5N5, and H5N6) with eight subclades (2.3.4.4a–2.3.4.4h) (14–16). In 2020, a novel reassortant HPAIV H5N1 clade 2.3.4.4b emerged in the Middle East and became the most predominant reassortant/variant in Asia, Europe, and Africa by 2021 (17). In December 2021, the HPAIV H5N1 was further spread via migratory birds across the Atlantic Ocean from Europe to North America, leading to several outbreaks among poultry populations (Fig. 1b) (18). Shortly thereafter, the HPAIV H5N1 clade 2.3.4.4b has reached Central and South America and has been frequently reported in farmed animals, several mammals, including humans and marines, and outdoor animals (13, 19–21). This wide host range and multiple cross-species infections with HPAIV H5N1 in wildlife and domestic animals has been recently expanded following the ongoing and unprecedented epizootic in dairy cattle (cows). Except for minks, marine mammals, and cows, infections of mammals with HPAIV H5N1 have not been documented to result in a mammal-to-mammal transmission (22–24). This highlights the importance of the three animal species, especially dairy cows, as potential factors in the emergence of HPAIV H5N1 variants that may transmit easily among human populations.

Until recently, cattle have been considered unlikely hosts for HPAIV H5N1 due to significant differences in their respiratory tract physiology compared to birds. However, a recent study demonstrated that avian SA-α2,3-Gal-β1,4, and SA-α2,3-Gal-β1,3 are present in the mammary gland, respiratory tract, and cerebrum of beef and/or dairy cattle. This finding provides a rationale for the observed propensity of HPAIV H5N1 to infect and replicate in the mammary glands (25). As the virus does not appear to infect cows efficiently via the respiratory route, most of the evidence points to cow-to-cow transmission via using the same milking devices that, once contaminated with infected milk, can inoculate the virus to the mammary gland of a non-infected cow during the milking process (26). Missing in this scenario is how the virus entered for the first time the mammary tissue of a cow, but one could speculate that perhaps a milking device was contaminated with HPAIV H5N1 from an infected bird, initiating the chain of cow-to-cow transmission.

Since its emergence in late 2020, HPAIV H5N1 clade 2.3.4.4b has caused worldwide epizootics in Europe, Africa, Asia, and the Americas (27, 28), and it has diversified into several genotypes circulating in several bird species, and showed the ability to infect more than 30 mammalian species with sporadic human cases (29). The origin of HPAIV H5N1 clade 2.3.4.4b is traced back as novel reassortant between H5N8 clade 2.3.4.4b and other IAV subtypes (30). Remarkably, HPAIV H5N1 clade 2.3.4.4b demonstrated substantiable geographic spread and host adaptation, facilitating its spread across various continents via migratory bird routes. By late 2021, the Eurasian HPAIV H5N1 clade 2.3.4.4b had reached North America through wild birds and subsequently been detected in a variety of mammalian species.

**Fig 2:**
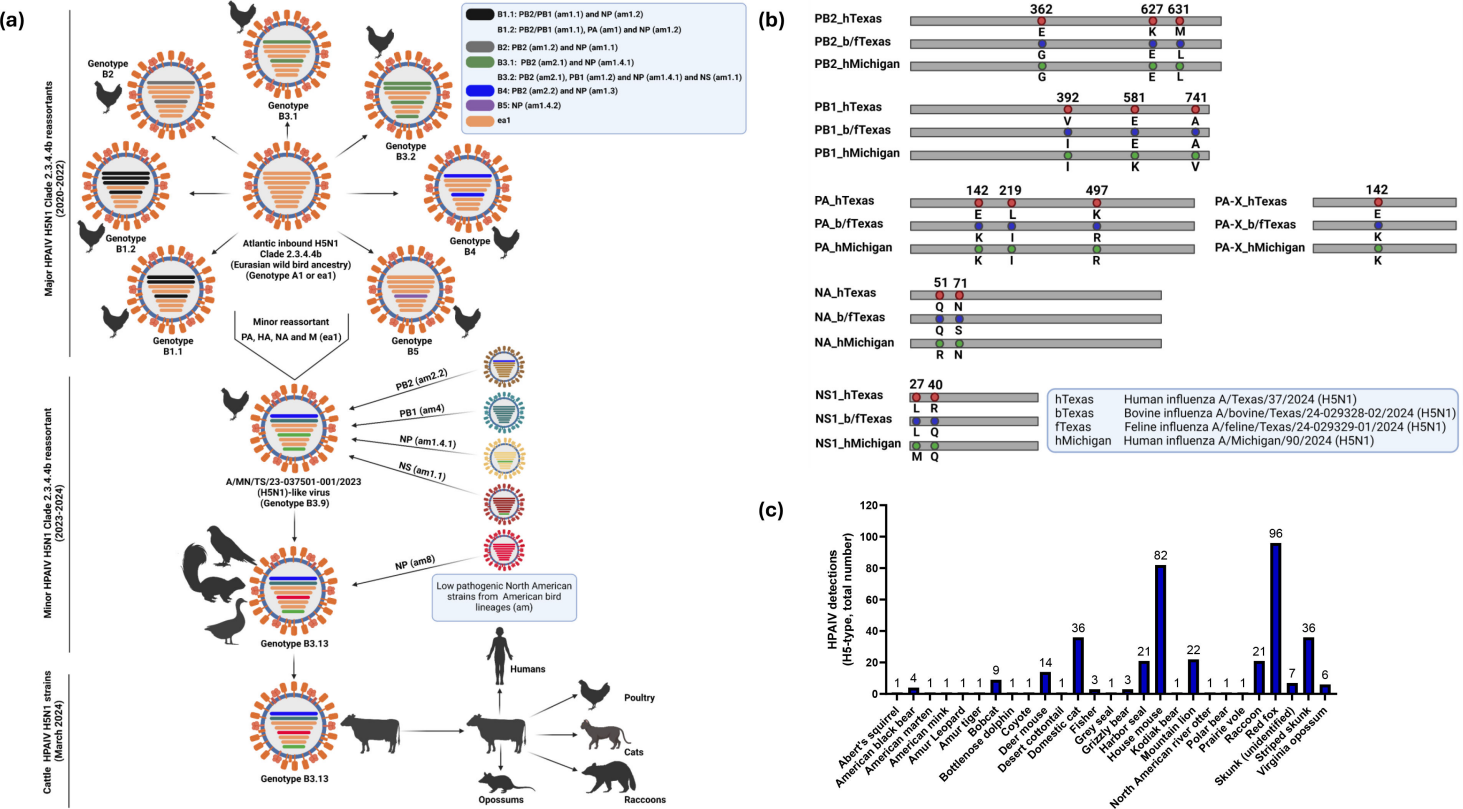
Genesis of the cattle-origin HPAIV H5N1 strain clade 2.3.4.4b genotype B3.13 and cross-species transmission: (a) Schematic representation of major H5N1 clade 2.3.4.4b reassortment events with the internal proteins encoding segments from North American low pathogenic strains and genesis of the reassortant cattle-origin HPAIV H5N1 strain clade 2.3.4.4b genotype B3.13. The genetic constellation of the seven major reassortants, frequently detected in poultry since the introduction of the ancestor Eurasian HPAIV H5N1 (genotype A1), were designated as B1.1, B1.2, B2, B3.1, B3.2, B4, and B5 (33). The predicted origin of internal proteins encoding segments from North American low pathogenic strains has been visualized in different colors that correspond the predicted ancestor. The minor reassortant B3.9 was further subject to a multiple reassortant events, acquiring the PA, HA, NA, and M segments from the genotype A1 and the other segments from co-circulating low pathogenic North American strains to generate avian HPAIV H5N1 of genotype B3.13 that has been emerged in November 2023 (24, 34). The avian HPAIV H5N1 of B3.13 genotype has further an NP segment reassortment event with another low pathogenic strain in unknown host to generate the first cattle HPAIV H5N1 strains of B3.13 genotype that were detected in human, cats, raccoons, and opossums in Texas in March/April 2024; (b) amino acid variations between closely related human, feline, and bovine isolates during the cattle HPAIV H5N1 outbreak in the United States. Human influenza A/Texas/37/2024 (H5N1) (hTexas, red), bovine influenza A/bovine/Texas/24-029328-02/2024 (H5N1) (bTexas, blue), feline influenza A/feline/Texas/24-029329-01/2024 (H5N1) (fTexas, blue), and human influenza A/Michigan/90/2024 (H5N1) (hMichigan, green) have identical HA, NP, M1, M2, and NEP. The amino acid variations between human and bovine/feline isolates in the polymerase subunits PB2, PB1, and PA and its by-product PA-X, NA, and NS1 proteins are indicated. The bTexas and fTexas isolates have identical amino acid sequences in all viral segments; (c) detections of HPAIV H5N1 in mammals in the United States from May 2022 to August 2024. Besides the HPAIV H5N1 detections in cattle in 2024 (Fig. 2b), the United States Department of Agriculture (USDA) has frequently reported infections with the HPAIV H5N1 clade 2.3.4.4b in 373 mammals representing 27 animal species in 32 states between May 2022 and August 2024. The figure has been created/assembled with BioRender.com.

In 2024, the first HPAIV H5N1 isolates from dairy cattle in Texas shared nearly identical genome sequences and were identified based on the phylogenetic analyses as clade 2.3.4.4b genotype B3.13, a reassortant of the minor B3.9 genotype reassortant of the Atlantic HPAIV H5N1 clade 2.3.4.4b (genotype A1) (24). This B3.13 genotype had emerged in late 2023 via reassortment between Eurasian wild bird H5N1 lineages (PA, HA, NA, and M gene segments) and American non-H5N1 wild bird lineages (PB2, PB1, NP, and NS gene segments) (Fig. 2a) (24, 31). Few weeks later, HPAIV H5N1 clade 2.3.4.4b genotype B3.13 was reported in several dairy farms in other localities of the United States, representing a multistate epizootic (32). Remarkably, the HPAIV H5N1 clade 2.3.4.4b genotype B3.13 reassortant virus implicated in the United States dairy cattle outbreak has never been detected so far in Europe. Similarly, no other Eurasian-North American reassortants have been recorded in Europe, despite detections of these viruses in Canada and the United States since 2014.

Different mutations in the HA and the polymerase subunits, which most likely facilitates viral entry and replication, respectively, in the new mammalian host cells, have been identified in dairy cattle HPAIV H5N1 (Table 1) (32). These mutations were found to enhance the binding affinity for sialic acid (SA) receptors prevalent in the mammalian upper respiratory tract, thereby enabling the virus to cross species and infect mammals. Additionally, alterations in the NA protein, which aids in the release of newly formed viral particles from infected host cells, have also been observed, most likely contributing to increased replication and transmission efficiency in cows (30–32). These changes might not only increase the ability of HPAIV H5N1 to establish infections in cattle but also may enhance its potential to spread from cattle to other mammals. This raises significant health concerns for both animals and humans.

**TABLE 1:** Amino acid variations, associated with increased mammalian host adaptation and pathogenicity, reported in HPAIV H5N1 isolated from cattle with frequency ≥90%

Gene	Amino acid mutation	Function	References
PB2	M631L	Human adaptive mutation that enhances polymerase activity and replication of mouse-adapted human and avian IAVs via adapting viral polymerases to use mammalian ANP32 proteins	(31, 35–37)
HA	172A	Enhances binding to α2–6 SA receptor without affecting the binding to α2–3 SA receptor	(31, 38)
	T199I	Contributes to decreased virion thermostability and increased HA activation pH and increases receptor binding breadth to mammalian- and avian-type sialic acid (SA) receptors via increasing receptor binding site flexibility	(31, 36, 39)
NP	V105M	Contribute to increased virulence, enhanced viral replication, severe pulmonary edema, and excessive inflammatory cellular infiltration in mammals	(31, 40, 41)
NA	N70S/D	Associated with decreased susceptibility of influenza infection to zanamivir	(42)
NA	N71S	Reported in immunocompromised patients with seasonal influenza infection that resist oseltamivir (E119V [100%]) after long-term treatment (139 days) with cumulative zanamivir therapy (E119V [100%] and N71S [100%])	(31, 43)
NS	V205I/G	Enhances the viral polymerase function and increases viral replication and lethality in mice	(44, 45)

In humans, the genetic analysis of the published sequences of human A/Texas/37/2024 (H5N1) (hereinafter referred to as hTexas) and A/Michigan/90/2024 (H5N1) (hereinafter referred to as hMichigan) to the closely related A/bovine/Texas/24-029328-02/2024 (H5N1) (hereinafter referred to as bTexas) or A/feline/Texas/24-029329-01/2024 (H5N1) (hereinafter referred to as fTexas) revealed that the hTexas isolate contains the well-known mammalian adaptive E627K mutation in the polymerase subunit PB2 (Fig. 2b). The emergence of PB2 E627K mutation in hTexas highlights the potential for the rapid evolution of these cattle HPAIV H5N1 post-infection. Likewise, hTexas is missing the PB2-M631L mammalian marker that exists in hMichigan, fTexas, and bTexas (Fig. 2b). The PB2-M631L is positively selected in 99% of HPAIV H5N1 isolates from dairy cattle and was found in the pre-cattle flu era to increase the capability of HPAIV H5N1 to replicate efficiently in human cells by enhancing their viral polymerase activity in mammalian cells (32, 46).

## CROSS-SPECIES TRANSMISSIONS OF HPAIV H5N1 CLADE 2.3.4.4B

Since January 2022, virus infections and outbreaks of HPAIV H5N1 clade 2.3.4.4b were documented in several mammalian species, including farmed mink in Spain, farmed foxes and other mammals in Finland (19, 47), sea lions in Peru, Argentina, and Chile (23), elephant seals in Argentina (48), domesticated pets such as cats in Poland, France, South Korea, and the United States (47), and dogs and cats in Italy (49). Since its introduction into the United States in 2022, the HPAIV H5N1 clade 2.3.4.4b expands its host range and was detected in 27 mammalian species (Fig. 2c). Most recently, the HPAIV H5N1 was detected in camelid cattle alpacas, baby goats, and dairy cattle in the United States (30).

**Fig 3:**
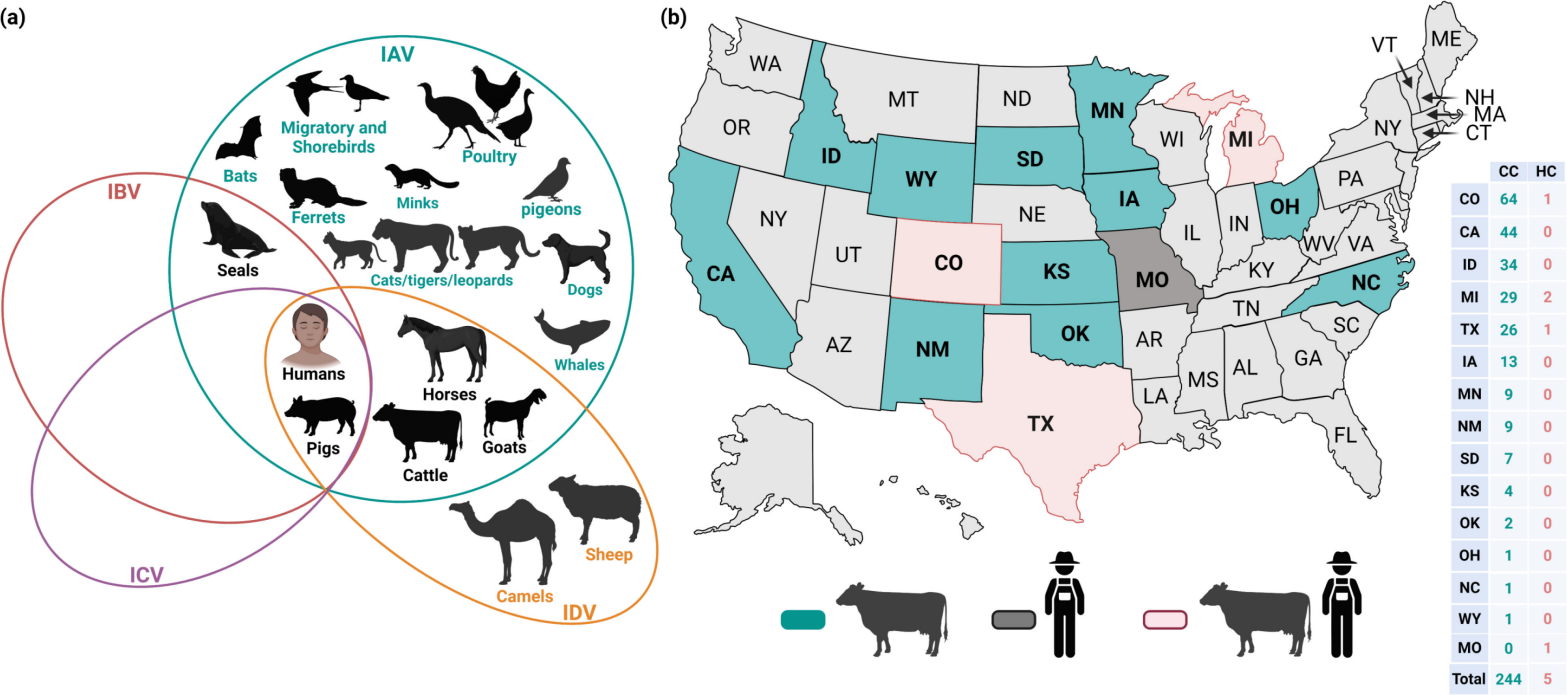
Updated host range of IAV, IBV, ICV, and IDV, and cattle H5N1 status in the United States: (a) documented hosts for influenza viruses show the broad-spectrum animal host species of IAV compared to IBV, ICV, and IDV; (b) confirmed cases of HPAIV H5N1 in dairy herds (CC) and human contact (HC) subjects. As of August 2024, HPAIV H5N1 has been detected in 189 dairy herds in 13 states (light blue, numbers between brackets represent the dairy herds affected). Three states, Colorado, Michigan, and Texas, have reported infections in dairy cattle as well as human infections in dairy herds’ workers (light red). The affected 13 states are Colorado (CO), Kansas (KS), Idaho (ID), Oklahoma (OK), Ohio (OH), New Mexico (NM), North Carolina (NC), Minnesota (MN), Michigan (MI), Iowa (IA), South Dakota (SD), Texas (TX), and Wyoming (WY). Numbers in the table indicate the cumulative numbers of dairy herd (CC) and human contact (HC) cases per affected state. The figure has been created/assembled with BioRender.com.

Ruminants, including dairy cattle and goats, were previously reported to be solely susceptible to efficient infections with IDV (50). Unlike IAV that recognizes either the avian-type α2–3-linked sialic acid receptor or mammalian-type α2–6-linked sialic acid receptor, IDV can infect cattle via binding of its hemagglutinin-esterase fusion protein into the 9-O-acetylated sialic acids receptors in cattle host cells (50, 51). Unlike IBV, ICV, and IDV, IAV, especially the HPAIV H5 subtype strains, displays broad host range, including avian, marine, and mammalian hosts (Fig. 3a). The recent detection of HPAIV H5N1 clade 2.3.4.4b viruses in ruminants, especially dairy cattle, goats, and other mammals such cats, minks, and sea lions (22, 47, 52–55), raises public health concerns about the epidemiology and evolution of this globally distributed virus with unusual expanded host range in non-avian hosts. Specially concerning has been its emergence in dairy cattle, where the virus has been efficiently transmitted among cows over several states of the United States.

Historically, the first documented IAV case in cattle was reported back in 1949 where 160,000 cattle were infected in Japan by H1 and H3 subtypes; however, no cattle-origin IAV was isolated from these outbreaks (56, 57). In 1953, Mitchell et al. demonstrated the ability of human IAV to persist in the mammary gland of cattle for 2 weeks (58). A follow-up study by Mitchell et al. in 1956 showed that IAV infection in the mammary gland is associated with virus detection in milk, and removal of mammary gland can result in a decrease in antibody content in blood (59). The first isolated IAV from cattle was documented in Hungary in the 1960s (60). Subsequently, few cases of IAV were identified in Russia in the 1970s–1980s. These cases were predominantly associated with outbreaks of respiratory disease in cattle, coinciding with the circulation of the H3N2 Hong Kong pandemic virus in humans (57). Despite that the transmission of seasonal IAV from humans to cattle has been documented in the past, these cattle seasonal IAVs could not adapt to establish efficient virus replication in cattle, sustained cattle-to-cattle or cattle-to-human transmission episodes, or virus evolution in cattle with bovine-specific adaptive genetic markers.

Prior to the ongoing H5N1 cattle outbreak, an experimental study documented the ability of HPAIV clade 2.2 strain A/cat/Germany/R606/2006 H5N1 to infect four calves intranasally via virus aerosolization (10^8.5^ EID_50_/mL) and house them with two uninfected contact calves (61). No or limited virus shedding was detected in nasal swabs. Nevertheless, all calves became seroconverted and remained healthy with no clinical signs (61). Most recently, two 2.3.4.4b HPAIV H5N1 representing the cattle-origin H5N1 B3.13 and avian origin European genotype euDG were experimentally used to infect calves via oronasal route (10^6^ TCID_50_/calf) and dairy cows via direct mammary gland inoculation (10^5.9^ TCID_50_/cow) (26). Interestingly, the calves presented mild respiratory signs of infection with moderate nasal replication and shedding. Notably, the cows presented severe mammary gland infection (no systemic infection), high fever, and reduced milk production with relatively high viral loads (26). Nevertheless the mammary gland infection of cows was not associated with systemic infection and no nasal shedding was detected in all infected cows (26). These data could further corroborate that the milk and milking procedures are contributing to the current cow-to-cow virus transmission. On the same hand, anti-H5N1 antibodies have been reported in sera collected from goats, buffalos, and cattle in Egypt (62). However, no natural infections or bird flu outbreaks were reported among cattle until the detection of the dairy cattle outbreak(s) in the United States on 25 March 2024 (Fig. 3b).

As of 1 October 2024, 14 states have declared the detection of HPAIV H5N1 in 242 dairy herds. The majority (197/244, 80.74%) of these cattle flu outbreaks were reported in five states: Texas (26 dairy herds affected, 10.66%), Michigan (29 dairy herds affected, 11.88%), Idaho (34 dairy herds and 1 alpaca herd affected, 13.93%), California (44 dairy herds affected, 18.03%), and Colorado (64 dairy herds affected, 26.23%). To date, 15 human cases of H5N1 have been reported in the United States, including five human cases that were associated with the current multistate outbreak of HPAIV H5N1 in dairy cattle between April and September 2024 (Fig. 3b). Four out of the five human cases had direct exposures to infected dairy cattle, but all of them have expressed mild-to-moderate symptoms including conjunctivitis (30). The recent fifth human case was reported from a person in Missouri with no known exposure to sick animals (63). The other 10 cases, including the reported 9 cases in 2024 in Colorado, were diagnosed H5-subtype positive following exposure to poultry that were infected with the cattle HPAIV H5N1. Since the detection of HPAIV H5N1 in cows, all H5N1 outbreaks in poultry in the United States as of the end of August are due to spillovers from infected cows.

The route of virus transmission into cattle is not clear yet, but as above discussed is likely to involve milking devices (26, 64). The cow-to-cow transmission and spread to several cattle farms in different states in the United States was confirmed and documented (65). By September 2024, five cattle flu-associated cases, including four farm workers in Colorado, Michigan, and Texas, were found positive for HPAIV H5N1 with conjunctivitis and mild respiratory symptoms (30, 66). The worker in the affected Texas dairy herd declared that he was in direct contact with HPAIV H5N1-infected cattle, wearing gloves but no face shield or eye protection goggles (30). This could support the hypothesis that the cattle-origin HPAIV H5N1 preferentially replicates in humans in the eye tissue following accidental virus transmission directly to the eye. This tissue tropism has also been observed in the limited number of human infections associated with previous outbreaks in chicken of avian H7 viruses (67). Virus transmission to cats, birds (e.g., blackbirds, pigeons, and common grackles), and other animals (e.g., raccoon and two opossums) was also reported and was confirmed by sequence genetic markers (e.g., PB2 M631L and PA K497R mutations) (32).

## PUBLIC HEALTH IMPLICATIONS AND CONTROL MEASURES

The route of transmission from infected dairy cattle to contact animals is not clear yet but was probably due to drinking of the infected unpasteurized milk or direct contact with contaminated/infected cattle or cattle milk. Infections of dairy cattle with HPAIV H5N1 were associated with mild-to-fatal clinical symptoms including high fever, respiratory distress, decreased rumination, and a significant drop in milk production and mortality (32, 66). Pasteurization effectively inactivates HPAIV H5N1, ensuring the safety of commercial milk supplies for human consumption. A recent study revealed that mice given raw milk samples from dairy cattle infected with HPAIV H5N1 exhibited lethal disease with high viral loads in their respiratory organs and mammary glands (68). Due to the high viral H5N1 titers achieved in milk from infected cows, individuals must refrain from consuming raw milk or raw milk by-products. Recently, researchers from the Center for Disease Control and Prevention (CDC) reported that the hTexas strain can infect ferrets leading to 100% mortality in all infected animals (*n* = 6) with efficient spread between ferrets that were in direct contact with infected ferrets. Moreover, the nontraditional ocular inoculation of hTexas into ferrets, even with a low dose of inoculum, was associated with severe and fatal infection (69). However, the hTexas virus exhibited limited transmission between ferrets via respiratory droplets (70).

Additionally, the HPAIV H5N1 clade 2.3.4.4b has been detected in 14 samples from deer mice in Colorado and 82 house mice in Colorado and New Mexico state since May 2024 (71). It is crucial to note that the HPAIV H5N1 can continue to undergo genetic mutations in this wide range of affected mammalian species that may enhance its transmissibility to and among humans. From a public health perspective, there is a necessity to discover why this HPAIV H5N1 clade 2.3.4.4b can currently infect this unusual wide range of mammalian species, including humans. Ongoing surveillance and sequence comparisons to monitor these H5N1 viruses in different hosts is imperative for better pandemic preparedness and are required to better understand spillover events and the underlying pathogenic mechanisms. In addition, functional assays to evaluate the role of the new amino acid changes are needed. But more important will be to eradicate the virus from cows to prevent further evolution and frequent contacts of H5N1 with humans. Current measures, including the need of a negative diagnostic test for IAV to transport cows from one farm to another, are not sufficient to quickly stop the outbreak in cows.

The HPAIV H5N1 genome was detected at high levels in the milk of affected dairy herds during the acute stage of infection (47). This issues public health alerts, suggesting the potential for the presence of infectious viral particles in unpasteurized milk. High levels of HPAIV replication in mammary tissues might be facilitated by the abundance of SA receptors in the mammary glands of dairy cattle, particularly avian influenza virus-specific SA-α2,3-gal (72). Mammary gland tissues from infected dairy cattle co-stained for SA and IAV nucleoprotein (NP) showed predominant co-localization of the virus and SA-α2,3-gal (47, 72).

In parallel to the human and animal surveillance systems, the CDC’s National Wastewater Surveillance System established a wastewater monitoring system to track the presence and spread of HPAIV H5N1. This can complement and compensate the limited capacity of regular surveillance systems. This approach has a limitation that it cannot distinguish the source of the detected genetics materials, whether they are from infected cattle, human, or other species (73). Recently, the CDC announced that from 7 to 20 July 2024, the H5-type virus was detected in the waste water of 303 sites in the United States (74).

AIVs, specifically HPAIV H5N1, can infect humans leading in 50% of the diagnosed infected cases to acute influenza disease and mortality (1, 75). This number is probably overestimated due to the lack of diagnosis of H5N1 in humans with mild or no symptoms. Nevertheless, almost all people do not have pre-existing antibodies or immune protection against HPAIV H5N1. The CDC regularly develops candidate vaccine viruses (CVV) against these pathogens as a pandemic preparedness strategy. For HPAIV H5N1, the CDC developed two CVV: influenza A/Astrakhan/3212/2020-like (IDCDC-RG71A) and A/American wigeon/South Carolina/22-000345-001/2021-like (IDCDC-RG78A) vaccine strains. Interestingly, the hTexas isolated from the Texas farm worker cross-reacts and could be efficiently neutralized with ferret antisera produced against these existing CVVs for H5-type clade 2.3.4.4b virus (73), indicating antigenicity matching. Moreover, the HA protein of the hTexas virus is closely related to existing A/H5-type clade 2.3.4.4b CVV (30, 73). Four (L104M, L115Q, T195I, V210A) or two (L115Q, T195I) amino acid variations were only distinguishing between the HA1 of hTexas and IDCDC-RG71A or IDCDC-RG78A CVV, respectively.

Despite that vaccination of animals has an indirect potential to protect humans against emerging zoonotic viral pathogens with remarkable spreading potential, there are no approved vaccines in the United States against HPAIV H5N1 for the dairy cattle yet. In August 2024, the USDA has launched a notice to the Center for Veterinary Biologics with the update on the first field safety study for a nonviable, non-replicating H5N1 clade 2.3.4.4b vaccine for dairy cattle (76). The current situation of HPAIV H5N1 infections in dairy cattle and subsequent sporadic transmission to humans and other mammalian and avian species highlights the urgent need for potential vaccination programs for dairy cattle to help in eradicating the virus from cows and mitigate the occurrence of any further zoonotic infections.

To date, two classes of Food and Drug Administration-approved anti-influenza drugs are recommended by the CDC to efficiently control influenza virus infections: NA inhibitors (NAIs), oseltamivir, zanamivir, and peramivir, and the cap-dependent endonuclease inhibitor (CENI), baloxavir marboxil (77). Despite that these drugs are still effective against AIV strains, including recent HPAIV H5N1, viruses with impaired or reduced susceptibility to available antiviral drugs could emerge upon acquisition of specific substitutions in the viral NA (e.g., I117T/V, I223T, S247R, S247N, H274Y, and N294S) or PA (e.g., E23G/K/R, A34R, A37T, I38T, and E199G) proteins, as reported (78–81). Recently, the sequences of the NA and the PA segments of 202 cattle HPAIV H5N1 strains from two different farms in Texas and Ohio states were investigated for presence of antiviral resistance markers against currently licensed NAI and the CENI. One strain comprises the NA-T438I substitution (N1 numbering) that has been previously shown to reduce HPAIV H5N1 inhibition by NAI zanamivir and peramivir, but not oseltamivir (82). All other strains do not carry known resistance markers to NAI or CENI (83).

## RECOMMENDATIONS AND PERSPECTIVES

Over just a century, four global pandemics were caused by new IAV strains originating from avian or mammalian hosts (1). The HPAIV H5N1 clade 2.3.4.4b has raised significant alarm due to its rapid global spread, efficient transmission to novel bird species, and frequent infection and outbreaks of diverse terrestrial and marine mammals (84), often exhibiting viral genome changes associated with mammalian host adaptation (23). Although ruminants were previously not considered as host species for HPAIV H5N1, the emergence of HPAIV H5N1 in dairy herds across several states of the United States is a significant cause for concern. However, the current risk to public health is still considered low by the authorities and the World Health Organization (WHO).

Because IAV is continually evolving, there is a real risk that new mammals could become permanent reservoirs for HPAIV, including the H5N1 subtype. Therefore, the WHO has highlighted the necessity of global proactive viral surveillance programs. It is also key to monitor and perform studies through a one-health approach to gain a better understanding of the factors driving the rapid spread and prevalence of HPAIV H5N1 in birds and other wild or domestic species, including new mammalian host species (85, 86). Genomic sequencing of viruses isolated from clinical samples is key for the detection of new genetic reassortments and mutations that may affect replication or transmissibility in mammals, including humans, or antiviral resistance. Moreover, functional studies addressing the biological importance of observed changes in the viral genome are highly important. Overall, these studies could allow to control and prevent disease propagation and future pandemics from occurring. But research and surveillance alone does not stop pandemic threats unless they lead to the implementation of mitigation measures and virus eradication from potential reservoirs. Despite the low risk considered at this moment for the novel bovine H5N1 viruses by WHO, this virus might find a new reservoir and further adapt to transmission in other mammal species, including humans. It is then important to devise and implement quickly policies for the containment and eradication of this virus from cattle. For example, more stringent quarantine measures for the movement and handling of milking cattle and milk combined with a thorough decontamination of cattle farms and milking devices are likely to help to contain this outbreak and eliminate the virus from cattle more quickly. Currently, there are no approved vaccines against HPAIV H5N1 for cattle or humans, and the seasonal flu vaccines do not provide protection or provide limited protection against AIVs, including the HPAIV H5N1 subtype (82, 83). However, if HPAIV H5N1 infection and potential transmission in cattle continue to occur, H5-subtype vaccination program for cattle could be necessary to limit the impact on the sector and new transmission routes to other mammalian species, including humans.

In summary, current outbreaks in dairy cattle in the United States have raised question on whether HPAIV H5N1 could be the next pandemic threat of disease X or the next flu pandemic. We are facing a critical time to understand and address this new threat before the virus continues evolving and adapting. Although it is always possible that the outbreak in cows dwindles by itself, especially if infected cows are non-susceptible to reinfection, ignoring or minimizing the threat of the continuous adaptation of this virus to new hosts exposed to infected cattle and infected milk could lead to severe consequences for animal and human health.

## References

[1] Mostafa A, Abdelwhab EM, Mettenleiter TC, Pleschka S. 2018. Zoonotic potential of influenza a viruses: a comprehensive overview. Viruses 10:497. doi:10.3390/v10090497 30217093 PMC6165440

[2] Liu R, Sheng Z, Huang C, Wang D, Li F. 2020. Influenza D virus. Curr Opin Virol 44:154–161. doi:10.1016/j.coviro.2020.08.004 32932215 PMC7755673

[3] Leibler JH, Abdelgadir A, Seidel J, White RF, Johnson WE, Reynolds SJ, Gray GC, Schaeffer JW. 2023. Influenza D virus exposure among US cattle workers: a call for surveillance. Zoonoses Public Health 70:166–170. doi:10.1111/zph.13008 36370131 PMC10099784

[4] White SK, Ma W, McDaniel CJ, Gray GC, Lednicky JA. 2016. Serologic evidence of exposure to influenza D virus among persons with occupational contact with cattle. J Clin Virol 81:31–33. doi:10.1016/j.jcv.2016.05.017 27294672

[5] Karakus U, Mena I, Kottur J, El Zahed SS, Seoane R, Yildiz S, Chen L, Plancarte M, Lindsay L, Halpin R, Stockwell TB, Wentworth DE, Boons G-J, Krammer F, Stertz S, Boyce W, de Vries RP, Aggarwal AK, García-Sastre A. 2024. H19 influenza A virus exhibits species-specific MHC class II receptor usage. Cell Host Microbe 32:1089–1102. doi:10.1016/j.chom.2024.05.018 38889725 PMC11295516

[6] Fereidouni S, Starick E, Karamendin K, Genova CD, Scott SD, Khan Y, Harder T, Kydyrmanov A. 2023. Genetic characterization of A new candidate hemagglutinin subtype of influenza A viruses. Emerg Microbes Infect 12:2225645. doi:10.1080/22221751.2023.2225645 37335000 PMC10308872

[7] Boivin S, Cusack S, Ruigrok RWH, Hart DJ. 2010. Influenza A virus polymerase: structural insights into replication and host adaptation mechanisms. J Biol Chem 285:28411–28417. doi:10.1074/jbc.R110.117531 20538599 PMC2937865

[8] van de Sandt CE, Kreijtz J, Rimmelzwaan GF. 2012. Evasion of influenza A viruses from innate and adaptive immune responses. Viruses 4:1438–1476. doi:10.3390/v4091438 23170167 PMC3499814

[9] Kanrai P, Mostafa A, Madhugiri R, Lechner M, Wilk E, Schughart K, Ylösmäki L, Saksela K, Ziebuhr J, Pleschka S. 2016. Identification of specific residues in avian influenza A virus NS1 that enhance viral replication and pathogenicity in mammalian systems. J Gen Virol 97:2135–2148. doi:10.1099/jgv.0.000542 27405649

[10] Khalil AM, Martinez-Sobrido L, Mostafa A. 2023. Zoonosis and zooanthroponosis of emerging respiratory viruses. Front Cell Infect Microbiol 13:1232772. doi:10.3389/fcimb.2023.1232772 38249300 PMC10796657

[11] WHO/OIE/FAO H5N1 Evolution Working Group. 2008. Toward a unified nomenclature system for highly pathogenic avian influenza virus (H5N1). Emerg Infect Dis 14:e1. doi:10.3201/eid1407.071681 PMC2600337 18598616

[12] Seo Y-R, Cho AY, Si Y-J, Lee S-I, Kim D-J, Jeong H, Kwon J-H, Song C-S, Lee D-H. 2024. Evolution and spread of highly pathogenic avian influenza A(H5N1) clade 2.3.4.4b virus in wild birds, South Korea, 2022-2023. Emerg Infect Dis 30:299–309. doi:10.3201/eid3002.231274 38215495 PMC10826760

[13] Lee D-H, Torchetti MK, Winker K, Ip HS, Song C-S, Swayne DE. 2015. Intercontinental spread of Asian-origin H5N8 to North America through Beringia by migratory birds. J Virol 89:6521–6524. doi:10.1128/JVI.00728-15 25855748 PMC4474297

[14] Lewis NS, Banyard AC, Whittard E, Karibayev T, Al Kafagi T, Chvala I, Byrne A, Meruyert Akberovna S, King J, Harder T, Grund C, Essen S, Reid SM, Brouwer A, Zinyakov NG, Tegzhanov A, Irza V, Pohlmann A, Beer M, Fouchier RAM, Akhmetzhan Akievich S, Brown IH. 2021. Emergence and spread of novel H5N8, H5N5 and H5N1 clade 2.3.4.4 highly pathogenic avian influenza in 2020. Emerg Microbes Infect 10:148–151. doi:10.1080/22221751.2021.1872355 33400615 PMC7832535

[15] Cui P, Shi J, Wang C, Zhang Y, Xing X, Kong H, Yan C, Zeng X, Liu L, Tian G, Li C, Deng G, Chen H. 2022. Global dissemination of H5N1 influenza viruses bearing the clade 2.3.4.4b HA gene and biologic analysis of the ones detected in China. Emerg Microbes Infect 11:1693–1704. doi:10.1080/22221751.2022.2088407 35699072 PMC9246030

[16] Tian J, Bai X, Li M, Zeng X, Xu J, Li P, Wang M, Song X, Zhao Z, Tian G, Liu L, Guan Y, Li Y, Chen H. 2023. Highly pathogenic avian influenza virus (H5N1) clade 2.3.4.4b introduced by wild birds, China, 2021. Emerg Infect Dis 29:1367–1375. doi:10.3201/eid2907.221149 37347504 PMC10310395

[17] Xie R, Edwards KM, Wille M, Wei X, Wong SS, Zanin M, El-Shesheny R, Ducatez M, Poon LLM, Kayali G, Webby RJ, Dhanasekaran V. 2023. The episodic resurgence of highly pathogenic avian influenza H5 virus. Nature 622:810–817. doi:10.1038/s41586-023-06631-2 37853121

[18] Caliendo V, Lewis NS, Pohlmann A, Baillie SR, Banyard AC, Beer M, Brown IH, Fouchier RAM, Hansen RDE, Lameris TK, Lang AS, Laurendeau S, Lung O, Robertson G, van der Jeugd H, Alkie TN, Thorup K, van Toor ML, Waldenström J, Yason C, Kuiken T, Berhane Y. 2022. Transatlantic spread of highly pathogenic avian influenza H5N1 by wild birds from Europe to North America in 2021. Sci Rep 12:11729. doi:10.1038/s41598-022-13447-z 35821511 PMC9276711

[19] Elsmo EJ, Wünschmann A, Beckmen KB, Broughton-Neiswanger LE, Buckles EL, Ellis J, Fitzgerald SD, Gerlach R, Hawkins S, Ip HS, et al.. 2023. Highly pathogenic avian influenza A(H5N1) virus clade 2.3.4.4b infections in wild terrestrial mammals, United States, 2022. Emerg Infect Dis 29:2451–2460. doi:10.3201/eid2912.230464 37987580 PMC10683806

[20] Sillman SJ, Drozd M, Loy D, Harris SP. 2023. Naturally occurring highly pathogenic avian influenza virus H5N1 clade 2.3.4.4b infection in three domestic cats in North America during 2023. J Comp Pathol 205:17–23. doi:10.1016/j.jcpa.2023.07.001 37586267

[21] Puryear W, Sawatzki K, Hill N, Foss A, Stone JJ, Doughty L, Walk D, Gilbert K, Murray M, Cox E, Patel P, Mertz Z, Ellis S, Taylor J, Fauquier D, Smith A, DiGiovanni RA Jr, van de Guchte A, Gonzalez-Reiche AS, Khalil Z, van Bakel H, Torchetti MK, Lantz K, Lenoch JB, Runstadler J. 2023. Highly pathogenic avian influenza A(H5N1) virus outbreak in New England seals, United States. Emerg Infect Dis 29:786–791. doi:10.3201/eid2904.221538 36958010 PMC10045683

[22] Agüero M, Monne I, Sánchez A, Zecchin B, Fusaro A, Ruano MJ, Del Valle Arrojo M, Fernández-Antonio R, Souto AM, Tordable P, Cañás J, Bonfante F, Giussani E, Terregino C, Orejas JJ. 2023. Highly pathogenic avian influenza A(H5N1) virus infection in farmed minks, Spain, October 2022. Euro Surveill 28:2300001. doi:10.2807/1560-7917.ES.2023.28.3.2300001 36695488 PMC9853945

[23] Leguia M, Garcia-Glaessner A, Muñoz-Saavedra B, Juarez D, Barrera P, Calvo-Mac C, Jara J, Silva W, Ploog K, Amaro L, Colchao-Claux P, Johnson CK, Uhart MM, Nelson MI, Lescano J. 2023. Highly pathogenic avian influenza A (H5N1) in marine mammals and seabirds in Peru. Nat Commun 14:5489. doi:10.1038/s41467-023-41182-0 37679333 PMC10484921

[24] Caserta LC, Frye EA, Butt SL, Laverack M, Nooruzzaman M, Covaleda LM, Thompson AC, Koscielny MP, Cronk B, Johnson A, Kleinhenz K, Edwards EE, Gomez G, Hitchener G, Martins M, Kapczynski DR, Suarez DL, Alexander Morris ER, Hensley T, Beeby JS, Lejeune M, Swinford AK, Elvinger F, Dimitrov KM, Diel DG. 2024. Spillover of highly pathogenic avian influenza H5N1 virus to dairy cattle. Nature 634:669–676. doi:10.1038/s41586-024-07849-4 39053575 PMC11485258

[25] Kristensen C, Jensen HE, Trebbien R, Webby RJ, Larsen LE. 2024. The avian and human influenza A virus receptors sialic acid (SA)-α2,3 and SA-α2,6 are widely expressed in the bovine mammary gland. bioRxiv. doi:10.1101/2024.05.03.592326 PMC11347012 39127127

[26] Halwe NJ, Cool K, Breithaupt A, Schön J, Trujillo JD, Nooruzzaman M, Kwon T, Ahrens AK, Britzke T, McDowell CD, et al.. 2024. H5N1 clade 2.3.4.4b dynamics in experimentally infected calves and cows. Nature. doi:10.1038/s41586-024-08063-y PMC11754106 39321846

[27] Bevins SN, Shriner SA, Cumbee JC, Dilione KE, Douglass KE, Ellis JW, Killian ML, Torchetti MK, Lenoch JB. 2022. Intercontinental movement of highly pathogenic avian influenza A(H5N1) clade 2.3.4.4 virus to the United States, 2021. Emerg Infect Dis 28:1006–1011. doi:10.3201/eid2805.220318 35302933 PMC9045435

[28] Nagy A, Černíková L, Stará M. 2022. A new clade 2.3.4.4b H5N1 highly pathogenic avian influenza genotype detected in Europe in 2021. Arch Virol 167:1455–1459. doi:10.1007/s00705-022-05442-6 35469095

[29] Fusaro A, Gonzales JL, Kuiken T, Mirinavičiūtė G, Niqueux É, Ståhl K, Staubach C, Svartström O, Terregino C, Willgert K, Baldinelli F, Delacourt R, Georganas A, Kohnle L, European Food Safety Authority, European Centre for Disease Prevention and Control, European Union Reference Laboratory for Avian Influenza. 2024. Avian influenza overview December 2023–March 2024. EFSA J 22:e8754. doi:10.2903/j.efsa.2024.8754 38550271 PMC10977096

[30] Uyeki TM, Milton S, Abdul Hamid C, Reinoso Webb C, Presley SM, Shetty V, Rollo SN, Martinez DL, Rai S, Gonzales ER, Kniss KL, Jang Y, Frederick JC, De La Cruz JA, Liddell J, Di H, Kirby MK, Barnes JR, Davis CT. 2024. Highly pathogenic avian influenza A(H5N1) virus infection in a dairy farm worker. N Engl J Med 390:2028–2029. doi:10.1056/NEJMc2405371 38700506

[31] Nguyen T-Q, Hutter C, Markin A, Thomas M, Lantz K, Killian ML, Janzen GM, Vijendran S, Wagle S, Inderski B, et al.. 2024. Emergence and interstate spread of highly pathogenic avian influenza A(H5N1) in dairy cattle. bioRxiv. doi:10.1101/2024.05.01.591751 40273240

[32] Oguzie JU, Marushchak LV, Shittu I, Lednicky JA, Miller AL, Hao H, Nelson MI, Gray GC. 2024. Avian influenza A(H5N1) virus among dairy cattle, Texas, USA. Emerg Infect Dis 30:1425–1429. doi:10.3201/eid3007.240717 38848249 PMC11210641

[33] Youk S, Torchetti MK, Lantz K, Lenoch JB, Killian ML, Leyson C, Bevins SN, Dilione K, Ip HS, Stallknecht DE, Poulson RL, Suarez DL, Swayne DE, Pantin-Jackwood MJ. 2023. H5N1 highly pathogenic avian influenza clade 2.3.4.4b in wild and domestic birds: introductions into the United States and reassortments, December 2021–April 2022. Virol (Auckl) 587:109860. doi:10.1016/j.virol.2023.109860 37572517

[34] Hu X, Saxena A, Magstadt DR, Gauger PC, Burrough E, Zhang J, Siepker C, Mainenti M, Gorden PJ, Plummer P, Li G. 2024. Highly pathogenic avian influenza A (H5N1) clade 2.3.4.4b virus detected in dairy cattle. bioRxiv. doi:10.1101/2024.04.16.588916:2024.04.16.588916

[35] Idoko-Akoh A, Goldhill DH, Sheppard CM, Bialy D, Quantrill JL, Sukhova K, Brown JC, Richardson S, Campbell C, Taylor L, Sherman A, Nazki S, Long JS, Skinner MA, Shelton H, Sang HM, Barclay WS, McGrew MJ. 2023. Creating resistance to avian influenza infection through genome editing of the ANP32 gene family. Nat Commun 14:6136. doi:10.1038/s41467-023-41476-3 37816720 PMC10564915

[36] Good MR, Ji W, Fernández-Quintero ML, Ward AB, Guthmiller JJ. 2024. A single mutation in dairy cow-associated H5N1 viruses increases receptor binding breadth. bioRxiv. doi:10.1101/2024.06.22.600211:2024.06.22.600211 PMC11685663 39737954

[37] Staller E, Carrique L, Swann OC, Fan H, Keown JR, Sheppard CM, Barclay WS, Grimes JM, Fodor E. 2024. Structures of H5N1 influenza polymerase with ANP32B reveal mechanisms of genome replication and host adaptation. Nat Commun 15:4123. doi:10.1038/s41467-024-48470-3 38750014 PMC11096171

[38] Cohen M, Fisher CJ, Huang ML, Lindsay LL, Plancarte M, Boyce WM, Godula K, Gagneux P. 2016. Capture and characterization of influenza A virus from primary samples using glycan bead arrays. Virol (Auckl) 493:128–135. doi:10.1016/j.virol.2016.03.011 PMC4860064 27031581

[39] Watanabe Y, Arai Y, Daidoji T, Kawashita N, Ibrahim MS, El-Gendy E-D, Hiramatsu H, Kubota-Koketsu R, Takagi T, Murata T, Takahashi K, Okuno Y, Nakaya T, Suzuki Y, Ikuta K. 2015. Characterization of H5N1 influenza virus variants with hemagglutinin mutations isolated from patients. mBio 6:e00081-15. doi:10.1128/mBio.00081-15 25852160 PMC4453573

[40] Liu Q, Chen H, Huang J, Chen Y, Gu M, Wang X, Hu S, Liu X, Liu X. 2014. A nonpathogenic duck-origin H9N2 influenza A virus adapts to high pathogenicity in mice. Arch Virol 159:2243–2252. doi:10.1007/s00705-014-2062-y 24696271

[41] Nguyen NM, Sung HW, Yun K-J, Park H, Yeo S-J. 2020. Genetic characterization of a novel North American-origin avian influenza A (H6N5) virus isolated from bean goose of South Korea in 2018. Viruses 12:774. doi:10.3390/v12070774 32709116 PMC7411716

[42] McKimm-Breschkin JL. 2013. Influenza neuraminidase inhibitors: antiviral action and mechanisms of resistance. Influenza Other Respir Viruses 7:25–36. doi:10.1111/irv.12047 23279894 PMC4942987

[43] Baz M, Abed Y, McDonald J, Boivin G. 2006. Characterization of multidrug-resistant influenza A/H3N2 viruses shed during 1 year by an immunocompromised child. Clin Infect Dis 43:1555–1561. doi:10.1086/508777 17109288

[44] Pu J, Wang J, Zhang Y, Fu G, Bi Y, Sun Y, Liu J. 2010. Synergism of co-mutation of two amino acid residues in NS1 protein increases the pathogenicity of influenza virus in mice. Virus Res 151:200–204. doi:10.1016/j.virusres.2010.05.007 20546807

[45] Patil A, Anhlan D, Ferrando V, Mecate-Zambrano A, Mellmann A, Wixler V, Boergeling Y, Ludwig S. 2021. Phosphorylation of influenza A virus NS1 at serine 205 mediates its viral polymerase-enhancing function. J Virol 95:02369–20. doi:10.1128/JVI.02369-20 PMC8094967 33408177

[46] Zhang X, Xu G, Wang C, Jiang M, Gao W, Wang M, Sun H, Sun Y, Chang K-C, Liu J, Pu J. 2017. Enhanced pathogenicity and neurotropism of mouse-adapted H10N7 influenza virus are mediated by novel PB2 and NA mutations. J Gen Virol 98:1185–1195. doi:10.1099/jgv.0.000770 28597818

[47] Burrough ER, Magstadt DR, Petersen B, Timmermans SJ, Gauger PC, Zhang J, Siepker C, Mainenti M, Li G, Thompson AC, Gorden PJ, Plummer PJ, Main R. 2024. Highly pathogenic avian influenza A(H5N1) clade 2.3.4.4b virus infection in domestic dairy cattle and cats, United States, 2024. Emerg Infect Dis 30:1335–1343. doi:10.3201/eid3007.240508 38683888 PMC11210653

[48] Uhart M, Vanstreels RET, Nelson MI, Olivera V, Campagna J, Zavattieri V, Lemey P, Campagna C, Falabella V, Rimondi A. 2024. Massive outbreak of influenza A H5N1 in elephant seals at Península Valdés, Argentina: increased evidence for mammal-to-mammal transmission. bioRxiv. doi:10.1101/2024.05.31.596774:2024.05.31.596774 PMC11555070 39528494

[49] Moreno A, Bonfante F, Bortolami A, Cassaniti I, Caruana A, Cottini V, Cereda D, Farioli M, Fusaro A, Lavazza A, Lecchini P, Lelli D, Maroni Ponti A, Nassuato C, Pastori A, Rovida F, Ruocco L, Sordilli M, Baldanti F, Terregino C. 2023. Asymptomatic infection with clade 2.3.4.4b highly pathogenic avian influenza A(H5N1) in carnivore pets, Italy, April 2023. Euro Surveill 28:2300441. doi:10.2807/1560-7917.ES.2023.28.35.2300441 37650905 PMC10472752

[50] Kwasnik M, Rola J, Rozek W. 2023. Influenza D in domestic and wild animals. Viruses 15:2433. doi:10.3390/v15122433 38140674 PMC10748149

[51] Liu R, Sreenivasan C, Yu H, Sheng Z, Newkirk SJ, An W, Smith DF, Chen X, Wang D, Li F. 2020. Influenza D virus diverges from its related influenza C virus in the recognition of 9-O-acetylated N-acetyl- or N-glycolyl-neuraminic acid-containing glycan receptors. Virol (Auckl) 545:16–23. doi:10.1016/j.virol.2020.02.007 PMC7174096 32174455

[52] Restori KH, Septer KM, Field CJ, Patel DR, VanInsberghe D, Raghunathan V, Lowen AC, Sutton TC. 2024. Risk assessment of a highly pathogenic H5N1 influenza virus from mink. Nat Commun 15:4112. doi:10.1038/s41467-024-48475-y 38750016 PMC11096306

[53] Rabalski L, Milewska A, Pohlmann A, Gackowska K, Lepionka T, Szczepaniak K, Swiatalska A, Sieminska I, Arent Z, Beer M, Koopmans M, Grzybek M, Pyrc K. 2023. Emergence and potential transmission route of avian influenza A (H5N1) virus in domestic cats in Poland, June 2023. Euro Surveill 28:2300390. doi:10.2807/1560-7917.ES.2023.28.31.2300390 37535471 PMC10401914

[54] Rimondi A, Vanstreels RET, Olivera V, Donini A, Lauriente MM, Uhart MM. 2024. Highly pathogenic avian influenza A(H5N1) viruses from multispecies outbreak, Argentina, August 2023. Emerg Infect Dis 30:812–814. doi:10.3201/eid3004.231725 38413243 PMC10977829

[55] Tomás G, Marandino A, Panzera Y, Rodríguez S, Wallau GL, Dezordi FZ, Pérez R, Bassetti L, Negro R, Williman J, Uriarte V, Grazioli F, Leizagoyen C, Riverón S, Coronel J, Bello S, Páez E, Lima M, Méndez V, Pérez R. 2024. Highly pathogenic avian influenza H5N1 virus infections in pinnipeds and seabirds in Uruguay: implications for bird-mammal transmission in South America. Virus Evol 10:veae031. doi:10.1093/ve/veae031 38756986 PMC11096771

[56] Saito K. 1951. An outbreak of cattle influenza in Japan in the fall of 1949. J Am Vet Med Assoc 118:316–319. 14832129

[57] Sreenivasan CC, Thomas M, Kaushik RS, Wang D, Li F. 2019. Influenza A in bovine species: a narrative literature review. Viruses 11:561. doi:10.3390/v11060561 31213032 PMC6631717

[58] Mitchell CA, Walker RV, Bannister GL. 1953. Further experiments relating to the propagation of virus in the bovine mammary gland. Can J Comp Med Vet Sci 17:218–222. 17648631 PMC1791540

[59] Mitchell CA, Walker RV, Bannister GL. 1956. Studies relating to the formation of neutralizing antibody following the propagation of influenza and Newcastle disease virus in the bovine mammary gland. Can J Microbiol 2:322–328. doi:10.1139/m56-037 13316626

[60] Lopez JW, Woods GT. 1984. Influenza virus in ruminants: a review. Res Commun Chem Pathol Pharmacol 45:445–462. 6390588

[61] Kalthoff D, Hoffmann B, Harder T, Durban M, Beer M. 2008. Experimental infection of cattle with highly pathogenic avian influenza virus (H5N1). Emerg Infect Dis 14:1132–1134. doi:10.3201/eid1407.071468 18598640 PMC2600352

[62] El-Sayed A, Prince A, Fawzy A, Abdou MI, Omar L, Fayed A, Salem M, Nadra-Elwgoud. 2013. Sero-prevalence of avian influenza in animals and human in Egypt. Pak J Biol Sci 16:524–529. doi:10.3923/pjbs.2013.524.529 24498821

[63] CDC. 2024. CDC confirms human H5 bird flu case in Missouri

[64] Neumann G, Kawaoka Y. 2024. Highly pathogenic H5N1 avian influenza virus outbreak in cattle: the knowns and unknowns. Nat Rev Microbiol 22:525–526. doi:10.1038/s41579-024-01087-1 39060613 PMC12720498

[65] USCDC. 2024. Current H5N1 bird flu situation in dairy cows

[66] Garg S, Reed C, Davis CT, Uyeki TM, Behravesh CB, Kniss K, Budd A, Biggerstaff M, Adjemian J, Barnes JR, Kirby MK, Basler C, Szablewski CM, Richmond-Crum M, Burns E, Limbago B, Daskalakis DC, Armstrong K, Boucher D, Shimabukuro TT, Jhung MA, Olsen SJ, Dugan V. 2024. Outbreak of highly pathogenic avian influenza A(H5N1) viruses in U.S. dairy cattle and detection of two human cases - United States, 2024. MMWR Morb Mortal Wkly Rep 73:501–505. doi:10.15585/mmwr.mm7321e1 38814843 PMC11152367

[67] Belser JA, Lash RR, Garg S, Tumpey TM, Maines TR. 2018. The eyes have it: influenza virus infection beyond the respiratory tract. Lancet Infect Dis 18:e220–e227. doi:10.1016/S1473-3099(18)30102-6 29477464 PMC6035055

[68] Guan L, Eisfeld AJ, Pattinson D, Gu C, Biswas A, Maemura T, Trifkovic S, Babujee L, Presler R, Dahn R, Halfmann PJ, Barnhardt T, Neumann G, Thompson A, Swinford AK, Dimitrov KM, Poulsen K, Kawaoka Y. 2024. Cow's milk containing avian influenza A(H5N1) virus - heat inactivation and infectivity in mice. N Engl J Med 391:87–90. doi:10.1056/NEJMc2405495 38785313 PMC12809964

[69] Belser JA, Sun X, Pulit-Penaloza JA, Maines TR. 2024. Fatal infection in ferrets after ocular inoculation with highly pathogenic avian influenza A(H5N1) virus. Emerg Infect Dis 30:1484–1487. doi:10.3201/eid3007.240520 38916793 PMC11210645

[70] USCDC. 2024. CDC reports A(H5N1) ferret study

[71] USDA. 2024. Detections of highly pathogenic avian influenza in mammals

[72] Nelli RK, Harm TA, Siepker C, Groeltz-Thrush JM, Jones B, Twu NC, Nenninger AS, Magstadt DR, Burrough ER, Piñeyro PE, Mainenti M, Carnaccini S, Plummer PJ, Bell TM. 2024. Sialic acid receptor specificity in mammary gland of dairy cattle infected with highly pathogenic avian influenza A(H5N1) virus. Emerg Infect Dis 30:1361–1373. doi:10.3201/eid3007.240689 38861554 PMC11210646

[73] Garg S, Reed C, Davis CT, Uyeki TM, Behravesh CB, Kniss K, Budd A, Biggerstaff M, Adjemian J, Barnes JR, Kirby MK, Basler C, Szablewski CM, Richmond-Crum M, Burns E, Limbago B, Daskalakis DC, Armstrong K, Boucher D, Shimabukuro TT, Jhung MA, Olsen SJ, Dugan V. 2024. Outbreak of highly pathogenic avian influenza A(H5N1) viruses in U.S. dairy cattle and detection of two human cases — United States, 2024. MMWR Morb Mortal Wkly Rep 73:501–505. doi:10.15585/mmwr.mm7321e1 38814843 PMC11152367

[74] USCDC. 2024. Influenza A virus wastewater data

[75] WHO. 2024. Cumulative number of confirmed human cases for avian influenza A(H5N1) reported to WHO, 2003-2024. Available from: https://cdn.who.int/media/docs/default-source/influenza/h5n1-human-case-cumulative-table/2024_feb_tableh5n1.pdf?sfvrsn=bccd8c23_1&download=true. Retrieved 26 Feb 2024.

[76] USDA. 2024. Field studies with nonviable, non-replicating veterinary vaccines targeting highly pathogenic avian influenza in Livestock. Available from: https://www.aphis.usda.gov/sites/default/files/notice24-13.pdf

[77] Govorkova EA, Takashita E, Daniels RS, Fujisaki S, Presser LD, Patel MC, Huang W, Lackenby A, Nguyen HT, Pereyaslov D, Rattigan A, Brown SK, Samaan M, Subbarao K, Wong S, Wang D, Webby RJ, Yen HL, Zhang W, Meijer A, Gubareva LV. 2022. Global update on the susceptibilities of human influenza viruses to neuraminidase inhibitors and the cap-dependent endonuclease inhibitor baloxavir, 2018-2020. Antiviral Res 200:105281. doi:10.1016/j.antiviral.2022.105281 35292289 PMC9254721

[78] Fusaro A, Zecchin B, Giussani E, Palumbo E, Agüero-García M, Bachofen C, Bálint Á, Banihashem F, Banyard AC, Beerens N, et al.. 2024. High pathogenic avian influenza A(H5) viruses of clade 2.3.4.4b in Europe-Why trends of virus evolution are more difficult to predict. Virus Evol 10:veae027. doi:10.1093/ve/veae027 38699215 PMC11065109

[79] Han J, Perez J, Schafer A, Cheng H, Peet N, Rong L, Manicassamy B. 2019. Influenza virus: small molecule therapeutics and mechanisms of antiviral resistance. Curr Med Chem 25:5115–5127. doi:10.2174/0929867324666170920165926 PMC8735713 28933281

[80] Hamza H, Shehata MM, Mostafa A, Pleschka S, Planz O. 2021. Improved in vitro efficacy of Baloxavir Marboxil against influenza A virus infection by combination treatment with the MEK inhibitor ATR-002. Front Microbiol 12:611958. doi:10.3389/fmicb.2021.611958 33679636 PMC7928405

[81] Hickerson BT, Petrovskaya SN, Dickensheets H, Donnelly RP, Ince WL, Ilyushina NA. 2023. Impact of Baloxavir resistance-associated substitutions on influenza virus growth and drug susceptibility. J Virol 97:e0015423. doi:10.1128/jvi.00154-23 37404185 PMC10373543

[82] Andreev K, Jones JC, Seiler P, Kandeil A, Turner JCM, Barman S, Rubrum AM, Webby RJ, Govorkova EA. 2024. Antiviral susceptibility of highly pathogenic avian influenza A(H5N1) viruses circulating globally in 2022–2023. J Infect Dis 229:1830–1835. doi:10.1093/infdis/jiad418 37770028 PMC11175693

[83] SJCEIRR. 2024. Antiviral susceptibility testing of highly pathogenic avian influenza A(H5N1) viruses isolated from dairy cattle in the United States, 2024

[84] Plaza PI, Gamarra-Toledo V, Euguí JR, Lambertucci SA. 2024. Recent changes in patterns of mammal infection with highly pathogenic avian influenza A(H5N1) virus worldwide. Emerg Infect Dis 30:444–452. doi:10.3201/eid3003.231098 38407173 PMC10902543

[85] Nohynek H, Helve OM. 2024. One health, many interpretations: vaccinating risk groups against H5 avian influenza in Finland. Euro Surveill 29:2400383. doi:10.2807/1560-7917.ES.2024.29.25.2400383 38904113 PMC11191420

[86] Giacinti JA, Robinson SJ, Sharp CM, Provencher JF, Pearl DL, Stevens B, Nituch L, Brook RW, Jardine CM. 2024. Assessing avian influenza surveillance intensity in wild birds using a one health lens. One Health 18:100760. doi:10.1016/j.onehlt.2024.100760 38832079 PMC11145394

